# Multi-subregional susceptibility patterns in gliomas using sub-voxel quantitative susceptibility mapping

**DOI:** 10.1186/s41747-026-00847-3

**Published:** 2026-10-26

**Authors:** Shanmei Zeng, Nengjin Zhu, Junqiao Wang, Liwei Mazu, Yingqian Huang, Zuqi Xia, Siyuan He, Jiajing Wu, Haodong Qin, Zhiyun Yang, Xuesong Liu, Hongjiang Wei, Jianping Chu

**Affiliations:** 1 Department of Radiology, The First Affiliated Hospital, Sun Yat-Sen University, No. 58 Zhongshan Road 2, 510080 Guangzhou, Guangdong China; 2 MR Research Collaboration Team, Siemens Healthineers Ltd., Shanghai, China; 3 Department of Neurosurgery, The First Affiliated Hospital, Sun Yat-Sen University, No. 58 Zhongshan Road 2, 510080 Guangzhou, Guangdong China; 4 School of Biomedical Engineering, Shanghai Jiao Tong University, Shanghai, 200030 China

**Keywords:** Cell proliferation, Glioma, Iron, Magnetic resonance imaging, Necrosis

## Abstract

**Objective:**

To evaluate glioma susceptibility patterns and their clinical/pathological correlations using a sub-voxel quantitative susceptibility mapping (QSM) technique that quantifies the paramagnetic (χ_para_) and diamagnetic (χ_dia_) components (APART-QSM).

**Materials and methods:**

We enrolled 120 pathologically confirmed glioma patients (*n* = 120; aged 47 ± 13 years, mean ± standard deviation; 51 women) retrospectively and 6 for histologic validation prospectively. χ_para_/χ_dia_ maps were generated. Subregions included peritumoral edema (PE), subdivided into four equidistant subregions, tumor parenchyma (TP), and necrosis, cystic degeneration (CD). Associations between multi-subregional susceptibility and clinicopathological parameters and a conditional inference tree (CIT) model for genotyping were analyzed.

**Results:**

Compared to contralateral normal-appearing tissue (CNAT), TP in high-grade and isocitrate dehydrogenase (IDH)-wild-type gliomas exhibited increased χ_para_ (*p* ≤ 0.044), low-grade and IDH-mutant gliomas lower χ_bulk_, χ_para_ and χ_dia_ (*p* ≤ 0.015). PE demonstrated lower susceptibility than CNAT (*p* < 0.001), with χ_para_/χ_dia_ initially decreasing then increasing with peritumoral distance (*p* ≤ 0.008). Necrosis showed elevated χ_para_/χ_dia_ (*p* ≤ 0.025), and CD in oligodendrogliomas displayed higher χ_dia_ than CNAT (*p* = 0.019). The CIT model (TP χ_dia_ and PE χ_para_) achieved 0.702 accuracy. Univariable analysis showed that higher TP χ_para_/χ_dia_ correlated with shorter survival (*p* < 0.001). In the prospective subgroup, TP χ_para_ correlated with iron content (*r* = 0.901, *p* = 0.014), TP χ_dia_/χ_para_ correlated with Ki-67 (*r* = 0.841, *p* = 0.036).

**Conclusion:**

APART-QSM revealed distinct, clinically relevant susceptibility profiles across glioma subregions with potential associations with genotyping and prognosis, providing biomarkers for iron content and tumor proliferation. Distance-associated susceptibility pattern in PE may reflect peritumoral microenvironmental properties.

**Key Points:**

***Question*** Gliomas exhibit complex histopathology with heterogeneous magnetic susceptibility. Effective preoperative evaluation of multi-subregional susceptibility patterns in gliomas facilitates refined tissue characterization and guides clinical treatment.

***Findings*** Glioma subregions showed clinically relevant susceptibility profiles, correlating with iron content, tumor proliferation, and revealing a distance-associated peritumoral edema pattern.

***Relevance statement*** Our study used the APART-QSM technique to characterize multi-subregional susceptibility patterns and pathological changes in different glioma types, revealing its potential association with glioma genotyping and prognosis.

**Graphical Abstract:**

**Figure:**
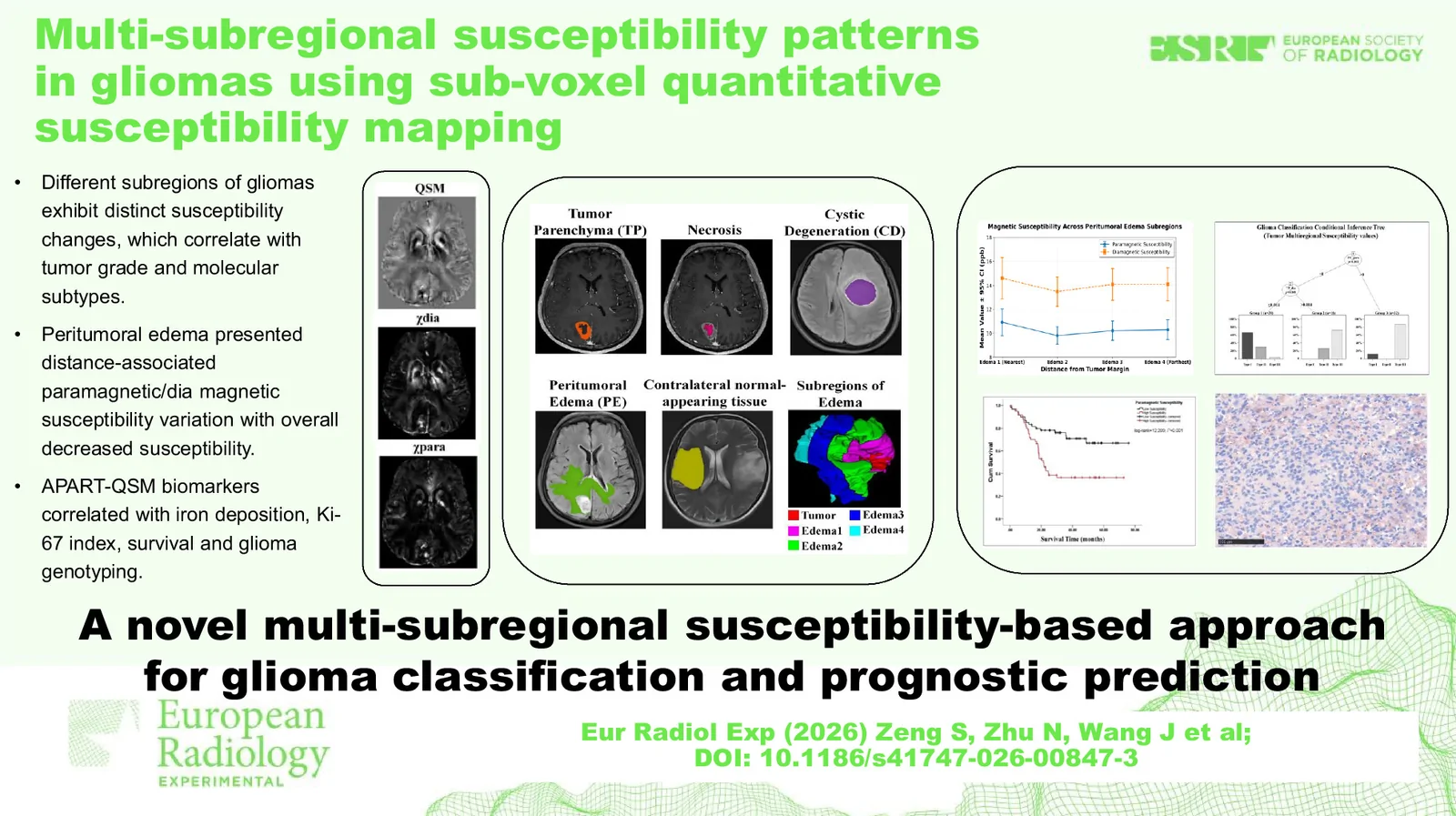

## Background

Diffuse gliomas are the most common primary brain tumors, with considerable mortality and morbidity [1, 2]. Their heterogeneous pathology manifests as notable variations in tissue magnetic susceptibility, particularly in high-grade gliomas (HGGs) [3]. Paramagnetic content increases due to factors such as hypoxia [4, 5] and elevated iron levels, the latter of which result from intratumoral hemorrhage [6], blood-brain barrier disruption [3], neovascularization [7–9], and aberrant iron metabolism [10]. Diamagnetic contributions arise from lipid accumulation related to tumor proliferation and calcification [3, 11]. Therefore, investigating magnetic susceptibility is crucial for understanding glioma pathology.

Glioma clinical relevance extends beyond the tumor parenchyma (TP). Necrosis and peritumoral edema (PE) are hallmarks of aggressive tumors, with PE correlating with infiltrating tumor cells beyond the main mass [12–14]. Additionally, cystic degeneration (CD) is frequent in oligodendrogliomas [15]. Consequently, multi-subregional susceptibility analysis likely provides more comprehensive clinical implications than whole-tumor assessment alone.

Quantitative susceptibility mapping (QSM) enables precise, quantitative measurement of tissue magnetic susceptibility [16–18]. Although prior studies have demonstrated the diagnostic value of QSM in glioma, most are limited by small sample sizes or insufficiently comprehensive analyses [3, 19, 20]. Critically, there remains a paucity of research investigating alterations in magnetic susceptibility across multiple distinct glioma subregions.

A fundamental limitation of conventional QSM is its measurement of voxel-averaged susceptibility, which cannot distinguish between intravoxel paramagnetic and diamagnetic components. This can lead to signal cancellation effects, especially in diffuse gliomas where complex constituents frequently coexist within individual voxels, thereby obscuring the true susceptibility profile. The recently developed sub-voxel QSM technique, IterAtive magnetic suscePtibility sources sepARaTion (APART-QSM), employs iterative data fitting to simultaneously separate and quantify paramagnetic and diamagnetic substances within a voxel, such as iron and myelination [21, 22]. APART-QSM has been applied in neurological disorders, including attention-deficit/hyperactivity disorder [23], epilepsy [24] and Alzheimer’s disease [25], providing *in vivo* biomarkers for pathological mechanisms. However, its potential for comprehensive glioma characterization remains largely unexplored.

Therefore, this study aims to comprehensively evaluate multi-subregional sub-voxel susceptibility alterations in glioma using APART-QSM, encompassing TP, necrosis, CD and PE. Furthermore, we analyzed the associations of these susceptibility metrics with tumor grade and molecular genotype, and further assessed their prognostic value for overall survival (OS). In addition, we examined spatial susceptibility variations across concentric PE subregions at increasing distances from the tumor margin and developed a conditional inference tree (CIT) model to classify gliomas into different genotypic subgroups based on their multi-subregional susceptibility profiles. Finally, we histologically validated the association between magnetic susceptibility and iron content within the TP in a prospectively enrolled subgroup.

## Materials and methods

### Patients

**Fig. 1:**
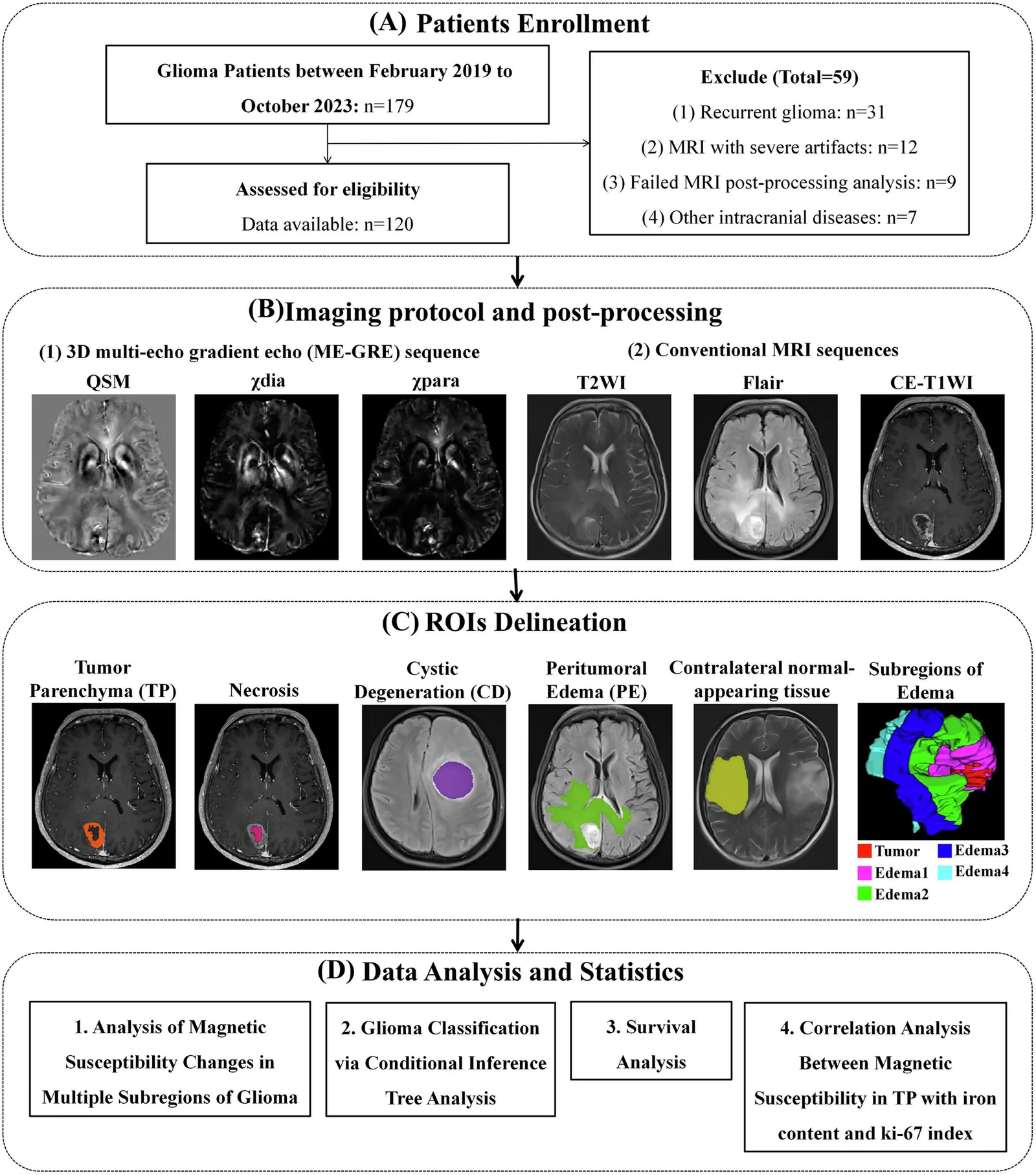
Study flowchart: **A** patient enrollment and exclusion criteria; **B** MRI acquisition and preprocessing; **C** delineation of multiple ROIs; **D** data analysis and statistical workflow. MRI, Magnetic resonance imaging; QSM, Quantitative susceptibility mapping; ROIs, Regions of interest

A total of 179 patients with pathologically confirmed gliomas who underwent preoperative MRI between February 2019 and October 2023 were initially screened. Following the exclusion criteria detailed in Fig. 1A, 120 patients were ultimately enrolled. Surgical resection was performed within two weeks after the MRI examination. OS tracked from surgery to death, or the last follow-up was obtained from clinical records or telephone interviews. Additionally, to further validate the pathologic basis of susceptibility measurements, we prospectively enrolled six patients with glioma and analyzed iron content and the Ki-67 proliferation index within the TP. The study was approved by the Institutional Review Board of the First Affiliated Hospital of Sun Yat-sen University (No. [2024]763). Approval was granted on 24 February 2024. Written informed consent was waived for 120 patients retrospectively enrolled, and written informed consent was obtained from 6 patients prospectively.

### MRI protocol and image preprocessing

Preoperative MRI was performed using a 3-T scanner (MAGNETOM Prisma, Siemens Healthcare), as illustrated in Fig. 1B. QSM was acquired prior to contrast administration using a three-dimensional multi-echo gradient-echo sequence. Detailed imaging parameters of QSM and conventional MRI sequences (T2-weighted, fluid-attenuated inversion-recovery (FLAIR) and T1-weighted magnetization-prepared rapid gradient-echo (MPRAGE) are provided in Appendix S1. APART-QSM comprised preprocessing (brain extraction, phase unwrapping, V-SHARP, STAR-QSM) and iterative separation (signal modeling, kernel regression, and alternating updates of susceptibilities and decay kernel until convergence). A two-level magnetic source estimation strategy was integrated into the APART-QSM framework to address susceptibility artifacts. The post-processing pipeline, including the APART-QSM method for reconstructing the different magnetic susceptibility maps, is also described in Appendix S1.

### Multi-subregional ROIs delineation

The QSM (χ_bulk_), paramagnetic (χ_para_) and diamagnetic (χ_dia_) maps, along with T2-weighted, FLAIR and contrast-enhanced T1-weighted MPRAGE images, were linearly coregistered using SPM12 in MATLAB R2021a. The ROIs including TP, PE, necrosis and CD regions were manually delineated in consensus by two experienced radiologists (with 12 and 10 years of experience in neuroradiology, respectively) to ensure the accuracy of all ROIs for subsequent analyses on axial contrast-enhanced T1-weighted MPRAGE, T2-weighted, and FLAIR images using ITK-SNAP software (www.itksnap.org), without the use of automated or semi-automated guidance (Fig. 1C). To assess interobserver reproducibility, a third neuroradiologist (with 8 years of experience), who was blinded to the consensus results, independently delineated the same regions. To analyze magnetic susceptibility alterations in PE at incremental distances from the tumor, the tumor mask was first progressively dilated outward using FMRIB Software Library (FSL, version 6.0; www.fmrib.ox.ac.uk/fsl) to obtain a series of dilated masks at incremental distances. For each dilated mask, the overlapping edema region was extracted, and the mean magnetic susceptibility of that region was calculated. The maximum extent of edema was defined as the point at which two consecutive dilations yielded identical susceptibility values within the overlapping edema mask, indicating that the dilated region had completely covered the entire edema area. The distance from the tumor margin to this maximal extent was then equally divided into four radial segments, thereby partitioning the total edema region into four equidistant concentric subregions radiating from the tumor border (from nearest to farthest: Edema1, Edema2, Edema3, Edema4) (Fig. 1C). Contralateral normal-appearing tissue (CNAT) masks were generated by mirroring the combined tumor and edema mask across the midline, and areas overlapping with the original lesion, ventricles, or extending beyond the brain were excluded (Fig. 1C).

### Molecular studies and iron content measurement

Immunohistochemistry and high-throughput sequencing were used to detect isocitrate dehydrogenase (IDH)1/2 mutations. The 1p/19q co-deletion status was determined by fluorescence *in situ* hybridization–FISH, with a signal ratio of 1p/1q < 0.70 and 19q/19p < 0.70 considered indicative of co-deletion.

For the prospective subset, intraoperative tumor specimens were collected. Iron deposition was evaluated using Perls’ Prussian blue staining on cryosections to correlate histological iron content with magnetic susceptibility values in the TP. The Ki-67 proliferation index was determined by immunohistochemistry.

### Statistical analysis

Statistical analysis was performed using SPSS (version 20.0). Data normality was assessed with the Kolmogorov–Smirnov test. Interobserver reliability for multi-subregional tumor measurements was evaluated using intraclass correlation coefficients based on a two-way random-effects model for absolute agreement. Paired comparisons between each subregion (TP, necrosis, CD, and PE) and their corresponding CNAT were performed using the Wilcoxon signed-rank test. A two-tailed Mann–Whitney *U-*test was used to compare two independent groups, including those across different tumor grades and molecular subtypes. Differences in magnetic susceptibility across PE subregions at varying distances from the tumor margin were assessed using repeated measures analysis of variance–ANOVA, with Bonferroni-adjusted post hoc comparisons. For the remaining subgroup comparisons, we report nominal *p*-values and interpret them cautiously without additional correction, to avoid overly conservative adjustments that may obscure potentially meaningful signals. For survival analysis, patients were dichotomized into high- and low-susceptibility groups based on the median value, and Kaplan-Meier curves were compared via the log-rank test. Univariable Cox proportional hazards regression was first performed separately for each multi-subregional susceptibility parameter. Subsequently, significant susceptibility and clinical variables were included in multivariate Cox regressions to identify the independent characteristics associated with the OS. Pearson’s correlation was used to assess the associations between susceptibility and iron content. Additionally, Spearman’s correlation was used to assess the associations between susceptibility and Ki-67 index. Statistical tests were two-sided, with significance defined as *p* < 0.05.

### Genotyping model construction

A glioma genotyping model was developed using CIT analysis to classify tumors into three types: IDH-mutant without 1p/19q co-deleted gliomas (Type Ⅰ), IDH-mutant and 1p/19q co-deleted gliomas (Type Ⅱ), and IDH-wildtype gliomas (Type Ⅲ). CIT analysis was performed using R software (version 4.4.2; R Foundation for Statistical Computing, Vienna, Austria) via the party package. Twelve multi-subregional susceptibility predictors were used, including χ_bulk_, χ_dia_ and χ_para_ from TP, PE, necrosis, and CD. The enrolled patients were randomly divided into training (60%, *n* = 73) and testing cohorts (40%, *n* = 47) using the caret package. To assess generalizability, a repeated tenfold cross-validation with 5 repeats was performed on the training set. Model performance was evaluated using accuracy with 95% confidence interval (CI), area under the receiver operating characteristic curve (AUC), sensitivity, specificity, and calibration curves.

## Results

### Patients

Patient demographics were summarized in Table 1. A total of 120 glioma patients were enrolled (aged 47 ± 13 years, mean ± standard deviation; range 19–70 years; 51 women). There were 59 IDH-mutant glioma patients, including 48 low-grade gliomas (LGGs) and 11 HGGs. There were 61 IDH-wildtype glioma patients, including 4 LGGs and 57 HGGs. 1p/19q co-deletion was present in 20 IDH-mutant and 1 IDH-wildtype glioma patients. PE was observed in 8 IDH-mutant and 45 IDH-wildtype patients. 16 IDH-mutant and 52 IDH-wildtype glioma patients showed necrosis. 20 IDH-mutant and 4 IDH-wildtype glioma patients showed CD. During follow-up, 54 patients died, and 66 were censored (including 7 lost to follow-up and 59 survivors).

**Table 1:** Participants demographics

Parameters	IDH mutation (*n* = 59)	IDH wildtype (*n* = 61)
Age (years, mean ± standard deviation)	40.76 ± 11.96	52.36 ± 12.43
Sex		
Male	31	38
Female	28	22
WHO classification		
II	35	4
III	13	1
IV	11	56
Glioma characteristics		
Necrosis	21	52
Cystic degeneration	20	4
Edema	8	45
1p/19q co-deletion	20	1
Follow-up outcome		
Death	9	45
Survival/loss to follow-up	47/3	12/4

Five IDH-wildtype glioma patients were classified as not otherwise specified due to the insufficient genotyping information

*IDH* Isocitrate dehydrogenase, *WHO* World Health Organization

### Interobserver reproducibility

Magnetic susceptibility measurements demonstrated excellent interobserver agreement across all tumor subregions (*p* < 0.001). The intraclass correlation coefficients of bulk, diamagnetic and paramagnetic susceptibility are presented in Appendix S2.

### Magnetic susceptibility alterations in TP

**Fig. 2:**
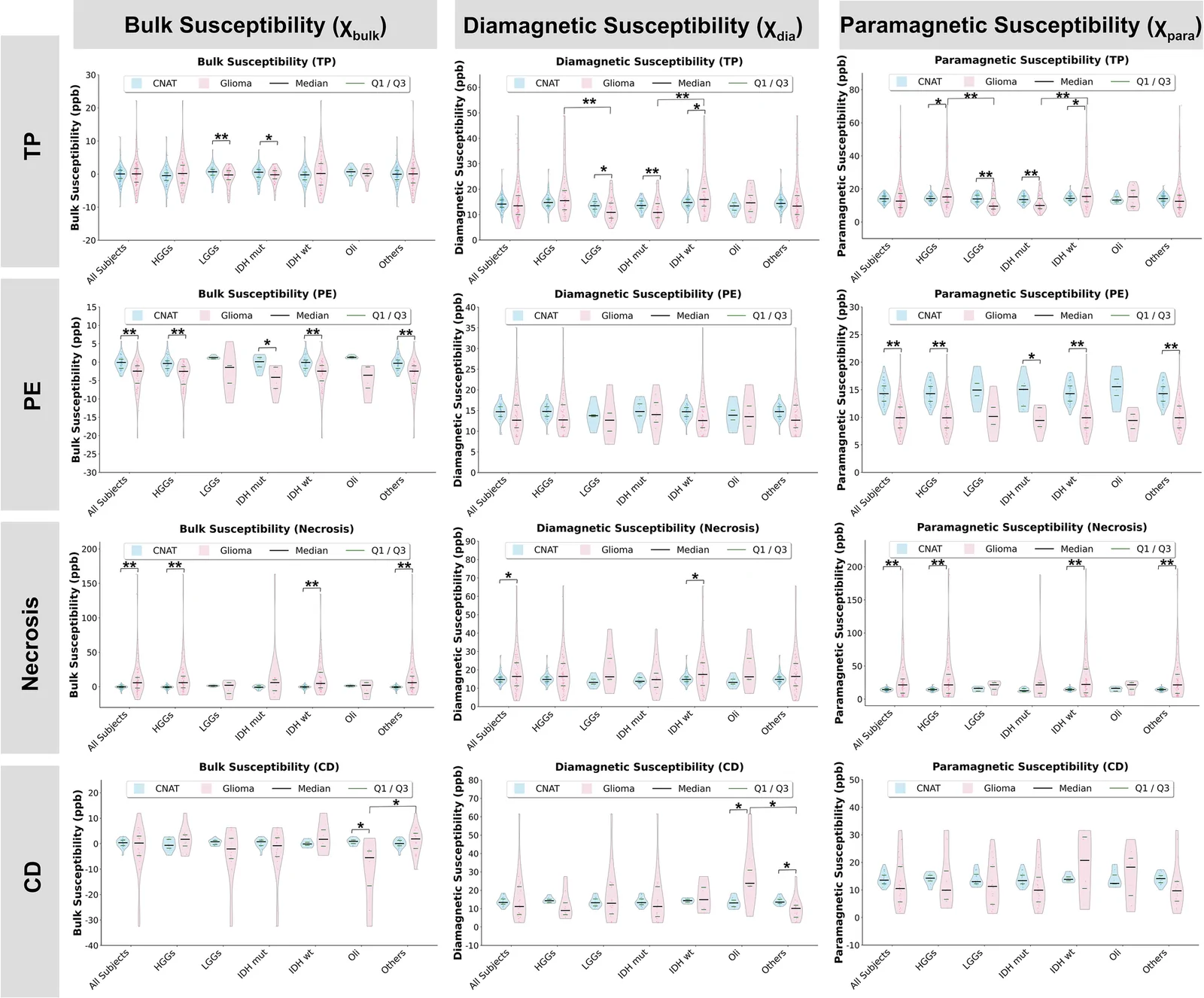
Violin plots of bulk magnetic susceptibility (QSM), paramagnetic susceptibility (χ_para_) and diamagnetic susceptibility (χ_dia_) across different glioma regions in different tumor grades, IDH mutations and 1p/19q co-deletion status. CD, Cystic degeneration; CNAT, Contralateral normal-appearing tissue; IDH, Isocitrate dehydrogenase; PE, Peritumoral edema; QSM, Quantitative susceptibility mapping; TP, Tumor parenchyma. * *p* < 0.05; ** *p* < 0.01

Compared with CNAT, TP in all gliomas showed higher bulk, paramagnetic and diamagnetic susceptibilities, although these differences were not statistically significant (Fig. 2).

HGGs exhibited significantly higher paramagnetic susceptibility in TP than CNAT (*p* = 0.044). Whereas, LGGs showed significantly lower bulk, paramagnetic and diamagnetic susceptibility (*p* ≤ 0.006). Besides, HGGs demonstrated significantly higher paramagnetic and diamagnetic susceptibility in TP than LGGs (*p* < 0.001) (Fig. 2).

IDH-mutated gliomas displayed significantly lower bulk, paramagnetic and diamagnetic susceptibility in TP than CNAT (*p* ≤ 0.015). Conversely, IDH-wildtype gliomas showed significantly elevated diamagnetic and paramagnetic susceptibility (*p* ≤ 0.034). IDH-wildtype gliomas exhibited significantly higher paramagnetic and diamagnetic susceptibility in TP than IDH-mutated gliomas (*p* < 0.001). No significant susceptibility differences were observed between TP and CNAT in oligodendrogliomas or non-oligodendrogliomas, nor between oligodendrogliomas and non-oligodendrogliomas (Fig. 2).

### Magnetic susceptibility alterations in PE

#### Overall evaluation

Compared with contralateral normal-appearing regions, PE in all gliomas demonstrated significantly lower bulk and paramagnetic susceptibility (*p* < 0.001) (Fig. 2). HGGs exhibited significantly lower bulk and paramagnetic susceptibility in PE than CNAT (*p* < 0.001). In contrast, no significant susceptibility differences were observed in PE of LGGs relative to CNAT, and susceptibility in PE did not differ significantly between HGGs and LGGs (Fig. 2). Both IDH-mutant and IDH-wildtype gliomas showed significantly lower bulk and paramagnetic susceptibility in PE *versus* CNAT (*p* ≤ 0.012 and *p* ≤ 0.001, respectively), with no significant difference between the two genotypes. In non-oligodendrogliomas, PE had significantly lower bulk and paramagnetic susceptibility than CNAT (*p* < 0.001). No significant differences in susceptibility were observed between PE and CNAT in oligodendrogliomas, nor between oligodendrogliomas and non-oligodendrogliomas (Fig. 2).

#### Association with distance in PE subregions

**Fig. 3:**
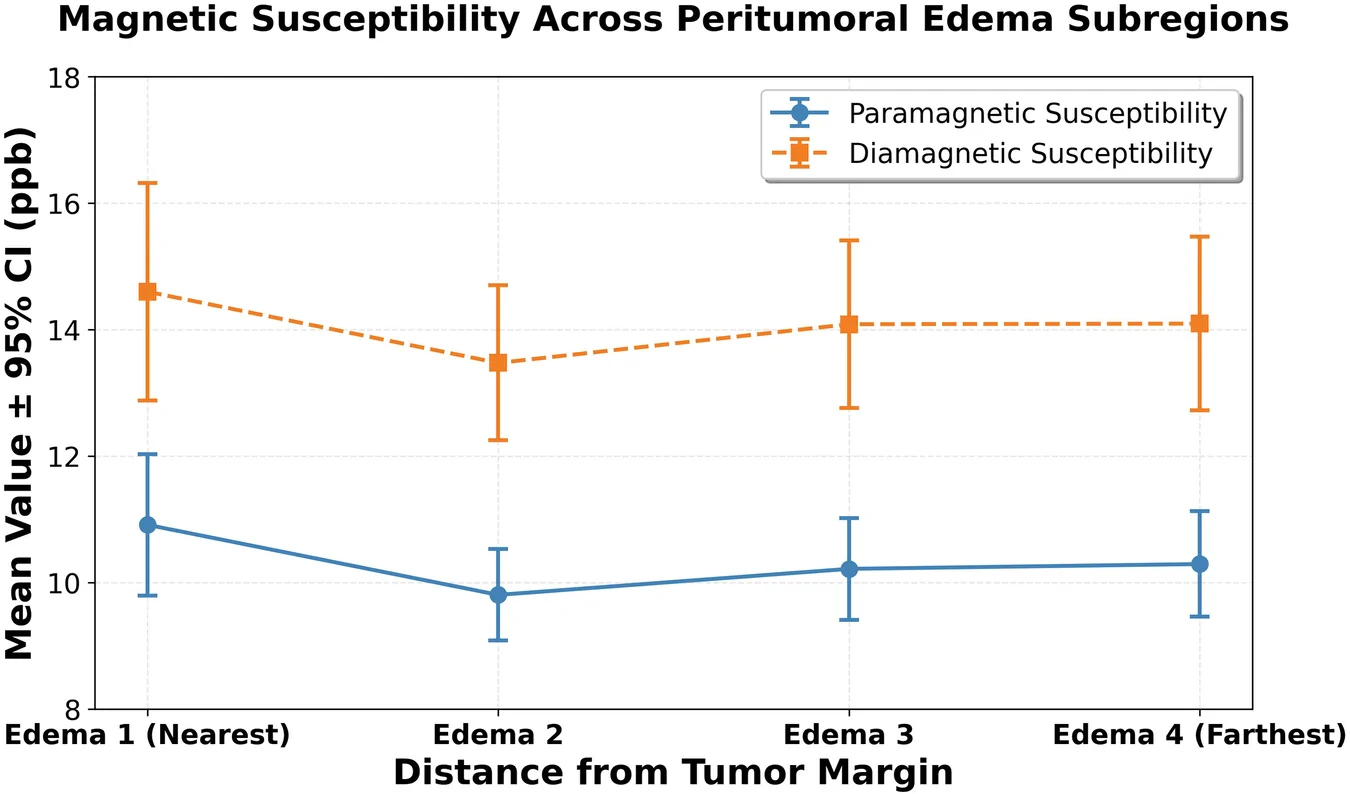
Line plots display the mean paramagnetic and diamagnetic susceptibility values within four concentric subregions (from Edema1 to Edema4) at increasing distances from the tumor margin

Analysis of PE subregions (edema1-edema4) revealed a significant association between susceptibility and distance from the tumor margin for both diamagnetic and paramagnetic components (*p* ≤ 0.008). Paramagnetic susceptibility was highest in nearest (edema1) (*p* ≤ 0.022, Bonferroni’s correction) and lowest in edema2 relative to all other subregions (*p* ≤ 0.005, Bonferroni’s correction) (Fig. 3). Similarly, diamagnetic susceptibility in edema1 was highest compared to the three more distal subregions, while only the difference between edema1 and edema2 reached statistical significance (*p* = 0.041, Bonferroni’s correction). Furthermore, diamagnetic susceptibility in edema2 was the lowest among all subregions, reaching statistical significance (*p* ≤ 0.041, Bonferroni’s correction) (Fig. 3).

### Magnetic susceptibility alterations in necrosis

Compared with CNAT, necrosis in all gliomas exhibited elevated bulk, diamagnetic, and paramagnetic susceptibility (*p* ≤ 0.025), and HGGs exhibited significantly higher bulk and paramagnetic susceptibility in necrosis (*p* < 0.001). No significant susceptibility alterations were observed in necrosis of LGGs, nor between HGGs and LGGs. Necrosis in IDH-wildtype gliomas exhibited increased bulk, paramagnetic and diamagnetic susceptibility (*p* ≤ 0.021), and non-oligodendrogliomas showed elevated bulk and paramagnetic susceptibility (*p* < 0.001), while IDH-mutant gliomas and oligodendrogliomas showed no significant changes relative to CNAT. Besides, no significant differences were found between different IDH mutation statuses, nor between oligodendrogliomas and non-oligodendrogliomas (Fig. 2).

### Magnetic susceptibility alterations in CD

Compared with CNAT, CD in all gliomas showed no significant difference in susceptibility, nor when stratified by tumor grade or IDH mutation status. However, in non-oligodendrogliomas, CD had significantly lower diamagnetic susceptibility (*p* = 0.019), while in oligodendrogliomas, CD showed lower bulk (*p* = 0.028) but higher diamagnetic susceptibility (*p* = 0.043) relative to CNAT. Besides, CD in oligodendrogliomas demonstrated significantly lower bulk and higher diamagnetic susceptibility than non-oligodendrogliomas (*p* ≤ 0.012) (Fig. 2).

### Glioma genotyping using CIT analysis based on multi-subregional magnetic susceptibility

**Fig. 4:**
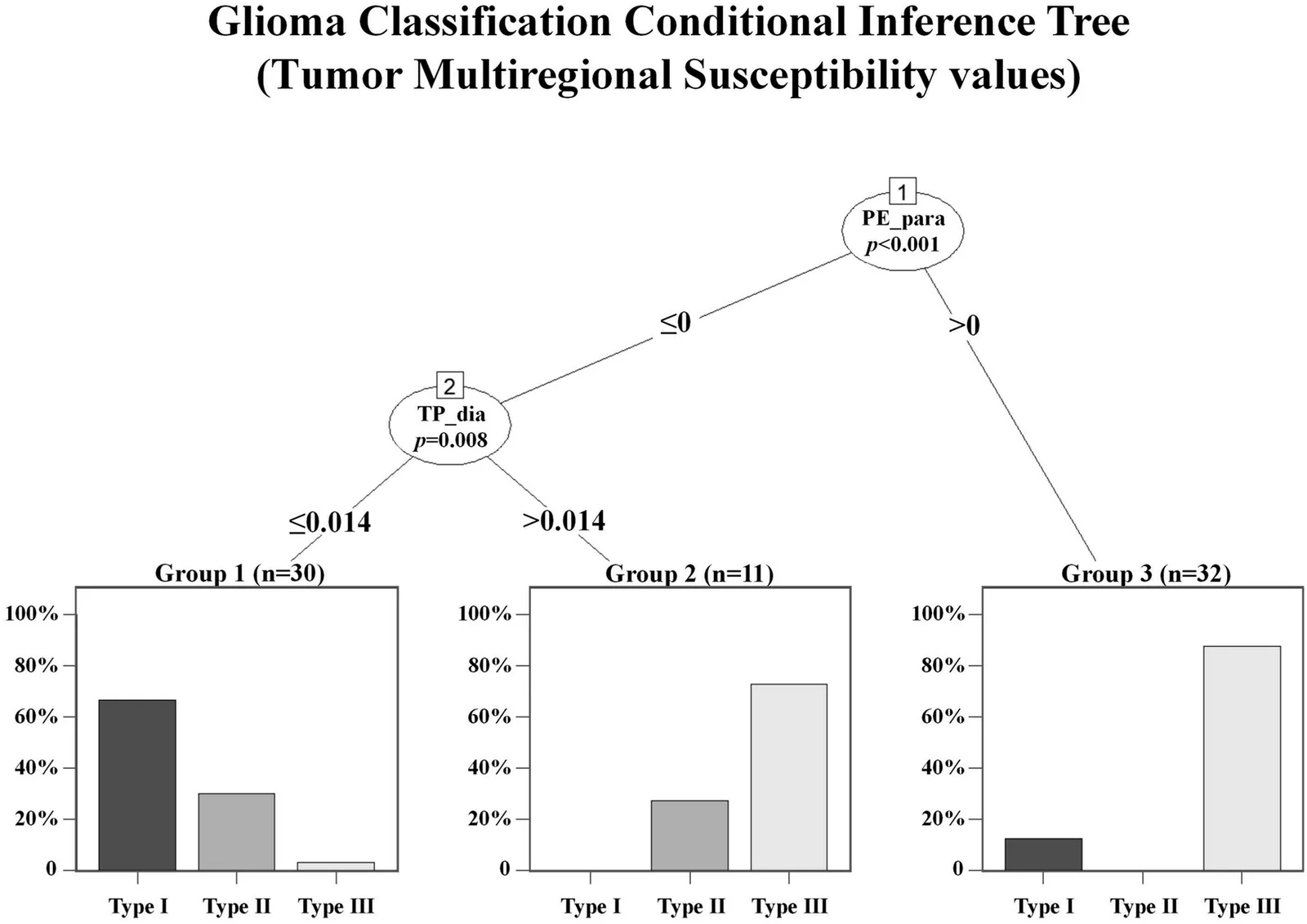
A conditional inference tree model plot for glioma genotyping based on multi-subregional susceptibility parameters. The model identifies diamagnetic susceptibility in TP and paramagnetic susceptibility in PE as the primary predictors. Group 1: PE χ_para_ < 0 and TP χ_dia_ ≤ 0.014; Group 2: PE χ_para_ < 0 and TP χ_dia_ > 0.014; Group 3: PE χ_para_ > 0. Type Ⅰ, IDH-mutant without 1p/19q co-deleted gliomas; Type Ⅱ, IDH-mutant with 1p/19q co-deletion gliomas; Type Ⅲ, IDH-wildtype gliomas; IDH, Isocitrate dehydrogenase; PE, Peritumoral edema; TP, Tumor parenchyma

The CIT model achieved an accuracy of 0.767 [95% CI: 0.670, 0.864] in the training set and 0.702 [0.571, 0.833] in the testing set for glioma classification. In training set, the AUC for identifying Type Ⅰ (IDH-mutant without 1p/19q codeleted gliomas) was 0.833 [0.749, 0.918], with a sensitivity of 83% and specificity of 80%; for Type Ⅱ (IDH-mutant with 1p/19q co-deletion gliomas), the AUC was 0.768 [0.674, 0.863], with a sensitivity of 0% and specificity of 100%; and for Type Ⅲ (IDH-wildtype gliomas), the AUC was 0.909 (0.839, 0.979), with a sensitivity of 97% and specificity of 81%. In the testing set, the corresponding AUCs were 0.851 [0.725, 0.977] with a sensitivity of 93% and specificity of 81% for Type Ⅰ, 0.619 [0.446, 0.791] with a sensitivity of 0% and specificity of 100% for Type Ⅱ, and 0.776 [0.648, 0.904] with a sensitivity of 79% and specificity of 65% for Type Ⅲ. The CIT analysis identified diamagnetic susceptibility within the TP and paramagnetic susceptibility in PE as significant predictors of molecular classification (Fig. 4). Detailed confusion matrices and calibration curves are provided in Appendix S3.

### Correlation of multi-subregional susceptibility with OS

**Fig. 5:**
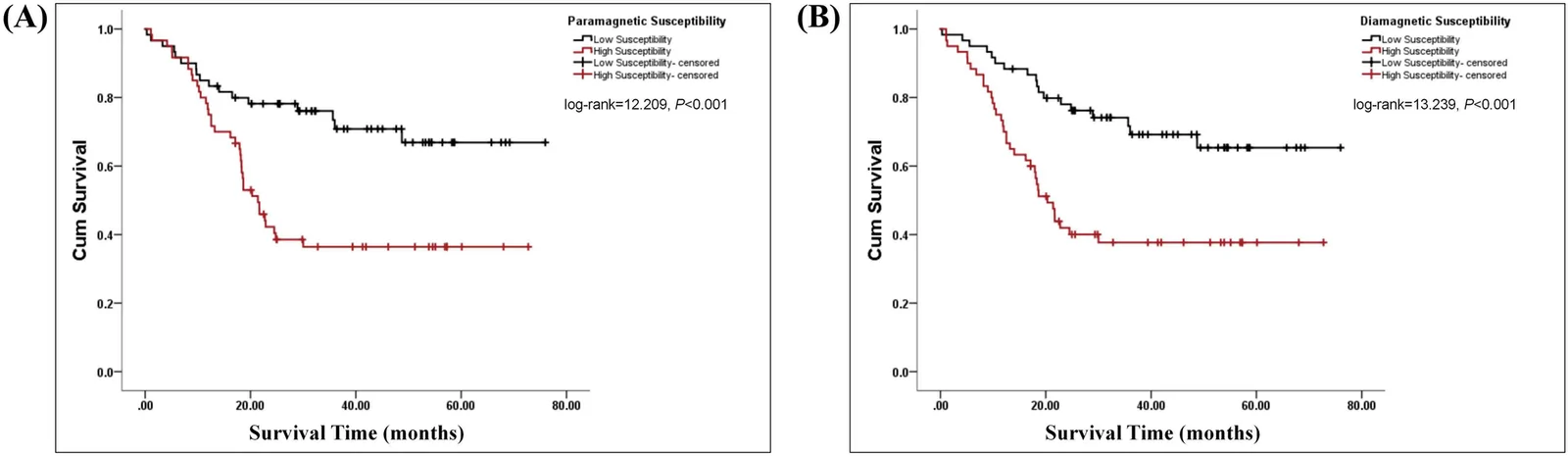
Kaplan–Meier survival curves demonstrated the association between magnetic susceptibility in tumor parenchyma and overall survival. Higher paramagnetic (**A**) and diamagnetic (**B**) susceptibility in tumor parenchyma were significantly associated with poorer overall survival

Kaplan-Meier analysis and univariate Cox regression show that the paramagnetic and diamagnetic susceptibility in TP were significantly associated with OS (paramagnetic susceptibility, log-rank = 12.209, *p* < 0.001, hazard ratio: 1.021, 95% CI: 1.003–1.040, *p* = 0.025; diamagnetic susceptibility, log-rank = 13.239, *p* < 0.001, hazard ratio: 1.037, 95% CI: 1.011–1.062, *p* = 0.004) (Fig. 5). However, multivariate Cox regressions showed only the age (hazard ratio: 1.035, 95% CI: 1.008–1.062, *p* = 0.001) was the independent prognostic factor.

### Correlation of magnetic susceptibility with iron content and tumor proliferation in TP

**Fig. 6:**
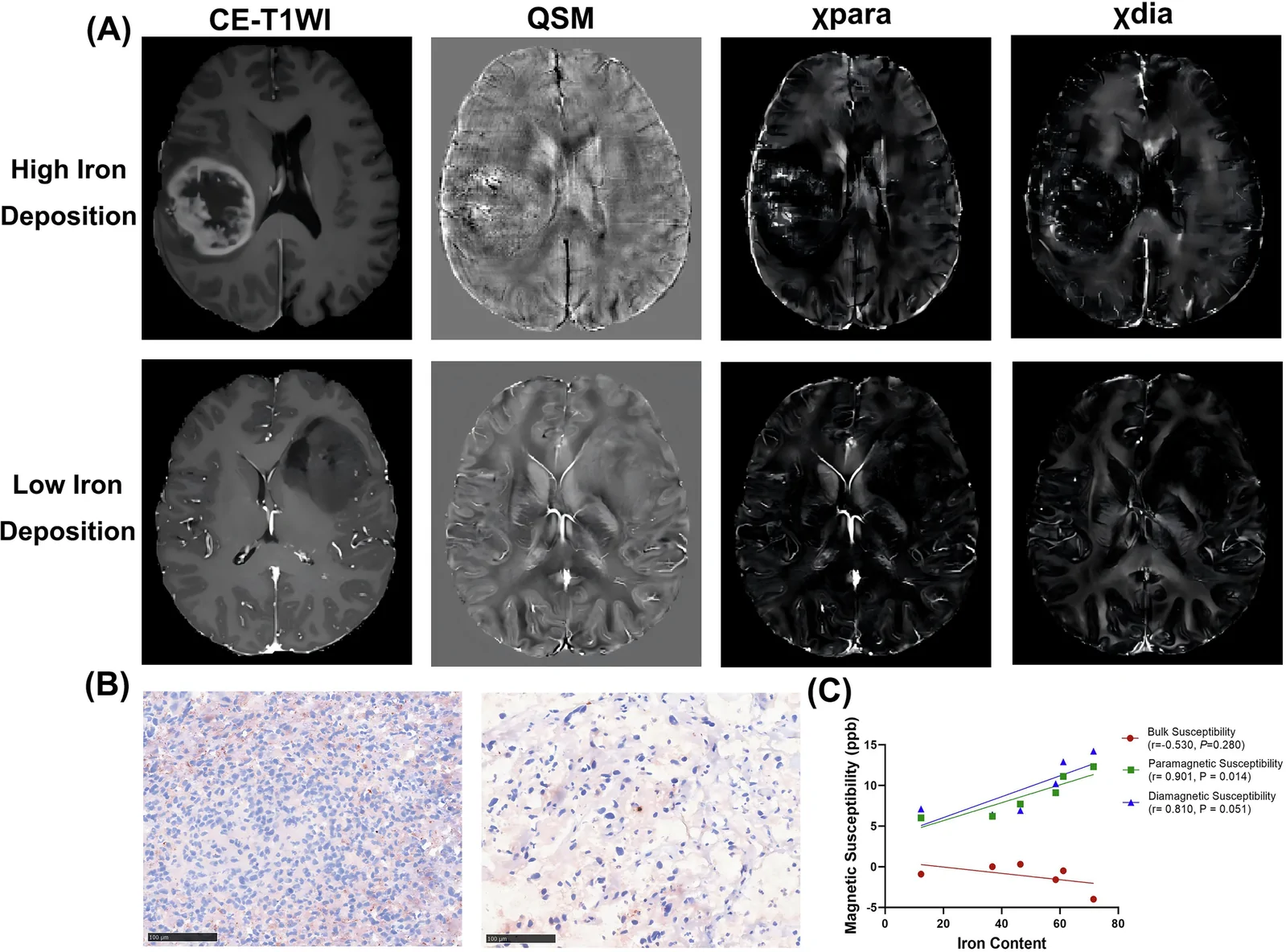
Correlation between magnetic susceptibility and histologic iron content in tumor parenchyma, based on the prospective subgroup (*n* = 6). **A** Representative axial slices (contrast-enhanced T1-weighted images, QSM, χ_para_, and χ_dia_ maps) from a patient with high iron deposition and a patient with low iron deposition. **B** Corresponding Perls’ Prussian blue staining (scale bar: 100 µm) confirming high and low iron content, respectively. **C** Scatter plots illustrating the correlations between magnetic susceptibility and iron content in tumor parenchyma. QSM, Quantitative susceptibility mapping

To validate the pathologic basis of susceptibility measurements, we performed correlation analyses in the prospectively enrolled subgroup (*n* = 6). A significant positive correlation was identified between iron deposition and paramagnetic susceptibility (*r* = 0.901, *p* = 0.014), though these preliminary findings warrant further validation in larger cohorts. No significant association was observed between iron content, bulk and diamagnetic susceptibility (Fig. 6).

To explore the effect of tumor proliferation on susceptibility measurements, we analyzed the association between Ki-67 and magnetic susceptibility in the same subgroup, and found both the paramagnetic (*r* = 0.841, *p* = 0.036) and diamagnetic (*r* = 0.841, *p* = 0.036) susceptibility correlated with Ki-67.

## Discussion

Our study leverages the sub-voxel quantitative capability of APART-QSM to systematically characterize magnetic susceptibility patterns across distinct glioma subregions, revealing significant associations with tumor grade, molecular genotype, and prognosis. Furthermore, we developed a CIT model based on multi-subregional susceptibility profiles for the classification of genotypic subgroups and validated the correlation between paramagnetic susceptibility and histologic iron content in TP. In contrast to conventional QSM and standard MRI sequences, our approach offers comprehensive susceptibility profiling across glioma subregions, providing novel insights into the pathological heterogeneity of gliomas and holding potential for refining noninvasive diagnostic paradigms.

In our exploratory subgroup analyses, we observed divergent susceptibility profiles in TP based on tumor grade and IDH status, which determine the degree of tumor aggressiveness. In more aggressive gliomas, the elevation in paramagnetic susceptibility may be attributable to increased intratumoral iron deposition, which could be associated with neovascularization, blood-brain barrier disruption, and altered iron metabolism [3, 6, 8, 10]. This interpretation is consistent with our preliminary observation of a positive correlation between paramagnetic susceptibility and histologic iron content in TP, though larger validation is needed. Additionally, hypoxia further enhances paramagnetic signals by increasing deoxyhemoglobin levels [4, 5, 26]. Concurrent elevation in diamagnetic susceptibility may be related to lipid accumulation associated with rapid tumor proliferation [3]. This interpretation is broadly consistent with our observed positive correlation between the Ki-67 index and diamagnetic susceptibility. Besides, the positive correlation between Ki-67 and paramagnetic susceptibility is likely due to enhanced intratumoral iron metabolism [10]. In contrast, less aggressive gliomas exhibit weaker dia- and paramagnetic susceptibility, likely attributable to their homogeneous tissue structure and elevated water content, consistent with homogeneous hyperintensity on T2-weighted images [27]. While our findings are consistent with those of a previous QSM-based study [28], our work provides a more detailed characterization of magnetic susceptibility alterations through a more comprehensive analysis.

In gliomas, PE is characterized by infiltrating tumor cells and inflammatory cells, the density of which generally decreases with increasing distance from the primary tumor, paralleling a reduction in the severity of edema [29, 30]. In our exploratory analysis of PE, the reduction of both the paramagnetic and diamagnetic values in PE may be attributed to elevated water content in peritumoral edema, causing a dilution effect on susceptibility signals [30], which attenuates measurable susceptibility changes. Interestingly, within the PE subregions, the paramagnetic and diamagnetic susceptibility followed a non-linear spatial pattern, which may reflect the dynamic interaction between infiltrating iron-rich tumor cells and inflammatory cells [31] and the degree of edema, which contains abundant weakly diamagnetic water [32]. A previous study has reported that the high magnetic susceptibility region in PE was associated with tumor infiltration and iron content [31]. Besides, as previously noted, lipids associated with tumor proliferation may contribute to increased diamagnetic susceptibility, and the interaction between these lipids and edema may underlie the observed non-linear spatial pattern.

Necrosis is characterized by thrombosis, hypoxia, blood-brain barrier disruption and neovascularization [13, 33], collectively increasing paramagnetic content. In our exploratory analysis of necrosis, elevated diamagnetic susceptibility in necrosis may arise from necrotic debris and denatured proteins, with underlying mechanisms to be further elucidated. Notably, necrosis in LGGs and IDH-mutant gliomas (predominantly oligodendrogliomas) showed no significant susceptibility alterations, likely due to small necrotic foci and limited sample size. Most CD-associated gliomas are low-grade, IDH-mutant tumors with high water content, leading to magnetic susceptibility comparable to CD itself. The higher diamagnetic susceptibility of CD in oligodendrogliomas was potentially due to intratumoral calcification [11], while non-oligodendrogliomas showed lower diamagnetic susceptibility, reflecting the predominance of weakly diamagnetic water in CD [32].

The CIT model identified diamagnetic susceptibility in TP and paramagnetic susceptibility in PE as key discriminative features for glioma genotyping, suggesting that subvoxel metrics from these regions may provide additional genotyping information than those derived from CD, necrosis, or conventional bulk susceptibility. However, it should be emphasized that the model is presented as an exploratory tool. Although it achieved a reasonable overall accuracy of 0.702, its performance was considerably limited for Type II gliomas. These findings are largely attributable to the data imbalance in our cohort (a limited number of oligodendroglioma cases), which also underscores the risk of overfitting to the minority subtype. Therefore, while the results are promising for Type I and Type III gliomas, the current model requires further validation in larger, more balanced cohorts.

Additionally, although both paramagnetic and diamagnetic susceptibilities in TP showed significant associations with shorter OS in univariable analysis, these associations did not remain significant after adjustment for other clinical covariates in multivariable Cox regression. This suggests that their prognostic value, while of potential interest, may be confounded by other clinical factors and that they require further validation in larger studies.

Our study had limitations. First, the exclusion of technically suboptimal cases may introduce selection bias, potentially limiting clinical generalizability. Standardized protocols and motion-robust techniques are needed in future studies. Second, the sample size was relatively modest, particularly for subgroups including oligodendrogliomas, tumors with CD or necrosis, and the histologic validation cohort. Larger cohorts and more balanced cohorts are needed in future studies. Third, the CIT model lacks external validation cohort, however, the current limited clinical implementation of QSM has restricted multi-center collaborations, future multi-institutional collaborations are needed to confirm and extend our model. Fourth, considering that rigorous correction could obscure meaningful signals, most subgroup analyses were performed using nominal, uncorrected *p*-values; therefore, these analyses should be interpreted as exploratory, and the findings warrant validation in independent cohorts. Additionally, although we examined the Ki-67–diamagnetic correlation, we did not directly validate the relationship between lipid accumulation/calcification and diamagnetic susceptibility, a relationship that warrants further investigation. Finally, the absence of pathological confirmation for susceptibility changes in PE subregions and necrosis constrains our mechanistic interpretation, underscoring the need for continued research.

## Conclusion

In conclusion, this exploratory study demonstrates that multi-subregional susceptibility analysis using APART-QSM reveals distinct, clinically relevant magnetic susceptibility profiles across glioma subregions that correlate with tumor grade and molecular genotype and show a suggestive association with patient prognosis. PE exhibits a unique potential distance-associated susceptibility pattern that may reflect peritumoral microenvironmental properties. These quantitative imaging biomarkers enable assessment of iron content and tumor proliferation and show promise for noninvasive molecular classification and prognostic relevance, supporting their integration into precision neuro-oncology frameworks.

## Supplementary information

**Additional file 1: Supplementary Figure 1.** Conditional inference tree model for glioma genotyping based on multi‑subregional susceptibility parameters. Confusion matrix for the training set (A) and the testing set (B). Calibration curves for the three glioma subtypes in the training set (C) and the testing set (D).

## Data Availability

Data generated or analyzed during the study are available from the corresponding author upon request.

## Abbreviations

APART-QSM: IterAtive magnetic suscePtibility sources sepARaTion QSM
AUC: Area under the receiver operating characteristic curve
CD: Cystic degeneration
CI: Confidence interval
CIT: Conditional inference tree
CNAT: Contralateral normal-appearing tissue
FLAIR: Fluid-attenuated inversion-recovery (FLAIR) and T1-weighted
HGGs: High-grade gliomas
IDH: Isocitrate dehydrogenase
LGGs: Low-grade gliomas
MPRAGE: Magnetization-prepared rapid gradient-echo
MRI: Magnetic resonance imaging
OS: Overall survival
PE: Peritumoral edema
QSM: Quantitative susceptibility mapping
TP: Tumor parenchyma
