# Supplementary material for: Multi-subregional susceptibility patterns in gliomas using sub-voxel quantitative susceptibility mapping
Source: Eur Radiol Exp. 2026 Oct 26;10:164. doi: 10.1186/s41747-026-00847-3 (PMC13710488; doi:10.1186/s41747-026-00847-3)
Supplement: Supplementary file 1 [file 41747_2026_847_MOESM1_ESM.pdf]

Multi-subregional susceptibility patterns in gliomas using sub-voxel quantitative susceptibility mapping

ELECTRONIC SUPPLEMENTARY MATERIAL

Eur Radiol Exp (2026) Zeng S, Zhu N, Wang J, et al.

Appendix S1

Materials and Methods

Imaging protocol

QSM

Quantitative susceptibility mapping (QSM) was performed prior to contrast agent administration using a three-dimensional multi-echo gradient echo (3D ME-GRE) sequence. Full brain coverage was obtained with the following imaging parameters: repetition time (TR), 35ms, echo time (TE) (7.50ms, 14.42 ms, 21.34 ms, 28.26 ms), field of view (FOV), 220 mm×220 mm; acquisition matrix, 416×288; number of slices, 32, slice thickness, 2mm, flip angle, 20°, voxel size, 0.5mm×0.5mm×2.0 mm, total acquisition time, 8 min 56 seconds.

Conventional MRI

The conventional MRI protocol comprised the following sequences: Transversal T2-weighted images: repetition time (TR)/ echo time (TE), 4200 ms/109 ms; field of view (FOV), 230 mm×230 mm; slice thickness, 6 mm; slice gap, 1.2 mm; Transversal T1-weighted images: repetition time (TR)/ echo time (TE), 2000 ms/17 ms; FOV, 230 mm×230 mm; slice thickness, 6 mm; slice gap, 1.2 mm. Axial fluid-attenuated inversion recovery images (FLAIR): TR/TE, 9000 ms/84 ms; FOV, 220 mm×220 mm; slice thickness, 6 mm; slice gap, 1.2 mm; A 3D-MPRAGE sequence (T1-MPRAGE) was acquired in the sagittal plane with TR/TE, 2300 ms/2 ms; section thickness with no intersection gap, 0.70 mm; FOV, 260 mm×260 mm. Contrast medium (0.1 mmol/kg body weight of Gd-DTPA, Magnevist, Schering, Berlin, Germany) was injected at a rate of 2mL/s, followed by a 20mL 0.9% saline flush at the same rate.

Magnetic susceptibility map reconstruction

The susceptibility maps were generated through the following post-processing pipeline. First, brain tissue was extracted from the GRE magnitude images to create a binary mask for subsequent processing, utilizing the Brain Extraction Tool (BET) in FSL. Phase images were processed using Laplacian-based phase unwrapping. Background field removal was then performed, and the tissue phase was extracted from the unwrapped phase using the V-SHARP method. An initial susceptibility map was subsequently reconstructed using the STAR-QSM algorithm.

Following this, the recently developed APART-QSM method was applied to reconstruct the paramagnetic and diamagnetic susceptibility maps. The APART-QSM employed a comprehensive complex signal model that describes the relationship between 3D GRE signal and the contributions of paramagnetic and diamagnetic susceptibility to the frequency shift and R_2^* relaxation:

$$S(TE_j, r) = M_0(r) e^{-(R_2(r) + a|\chi_{para}(r)| + a|\chi_{dia}(r)|) TE_j} \cdot e^{i\{\phi_{res}(r) + 2\pi f_{bg}(r) TE_j + 2\pi\gamma ¿} 0[D*(\chi_{para}(r) + \chi_{dia}(r))]E_j\} ¿¿$$

Where r is the position, M_0 is the extrapolated magnitude signal at TE = 0 ms, χ_{para} is the paramagnetic susceptibility, χ_{dia} is the diamagnetic susceptibility, ϕ_{res} represents the time-independent residual phase, $2\pi f_{bg} TE_j$ is the background phase, D is the magnetic dipole kernel , * denotes the spatial convolution, j is the index of echo times, and α is the magnitude decay kernel, representing the proportionality constant between $R_2' = (R^* - R_2)$ and absolute susceptibility sources.

R_2^* was assumed to be approximately proportional to the magnitude of susceptibility sources in the approximation of small concentrations. In this study, the approximated R_2' can be calculated according to the following equation:

$$R_2^* = \alpha R_2' + \beta$$

Here, α and β are the slope and intercept estimated from the linear least-squares regression between mean R_2^* and R_2' value using a linear model to represent the magnitude of susceptibility source concentration and R_2^*. With the approximated R_2', APART-QSM can reconstruct paramagnetic and diamagnetic susceptibility maps based on the GRE data. APART-QSM alternately estimated the magnitude decay kernel and the reconstructed susceptibilities in an iterative fitting fashion for each voxel, preventing the errors of parameter α from propagating to the final susceptibility separation reconstruction, thereby improving the reliability of the resulting susceptibility maps.

Appendix S2

Results

Interobserver Reproducibility

The ICC of bulk, diamagnetic and paramagnetic susceptibility were as follows: 0.89 [0.78-0.95], 0.98 [0.96-0.99] and 0.97 [0.94-0.99] in TP; 0.95 [0.89-0.98], 0.97 [0.93-0.99] and 0.95 [0.90-0.98] in necrosis; 0.94 [0.77-0.98], 0.92 [0.70-0.98] and 0.74 [0.27-0.93] in CD; 0.80 [0.60-0.91], 0.90 [0.78-0.97] and 0.73 [0.46-0.88] in PE.

Appendix S3

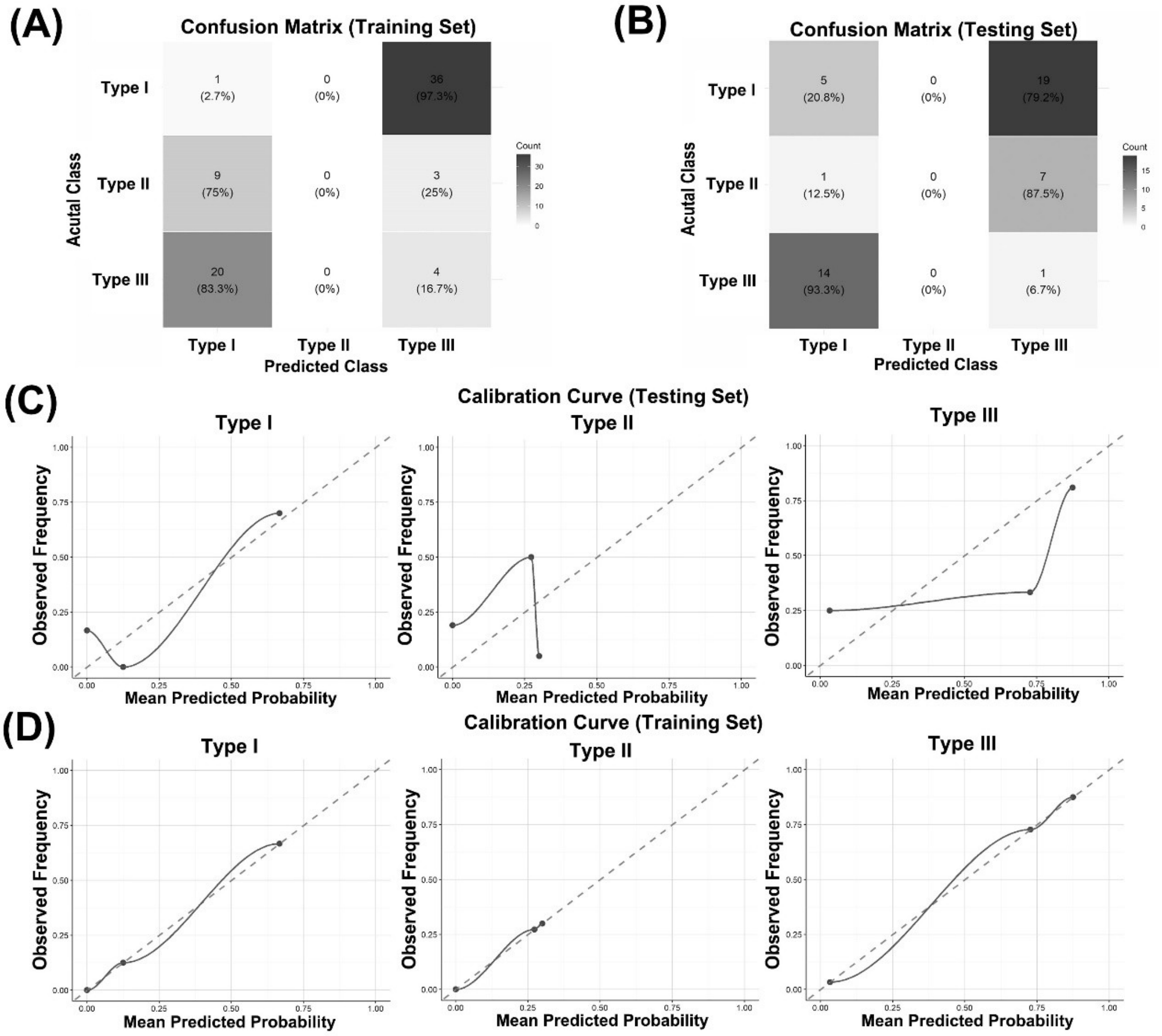

Supplementary Figure 1. Conditional inference tree model for glioma genotyping based on multi-subregional susceptibility parameters. Confusion matrix for the training set (A) and the testing set (B). Calibration curves for the three glioma subtypes in the training set (C) and the testing set (D).
